# Supplementary material for: Chromatin Remodelling Molecule ARID1A Determines Metastatic Heterogeneity in Triple-Negative Breast Cancer by Competitively Binding to YAP
Source: Cancers (Basel). 2023 Apr 25;15(9):2447. doi: 10.3390/cancers15092447 (PMC10177047; doi:10.3390/cancers15092447)

Supplement3 A

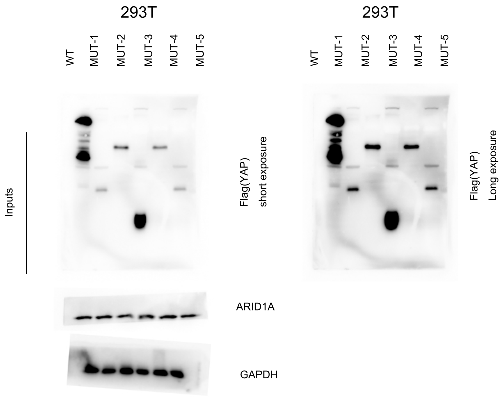

Supplement3 B

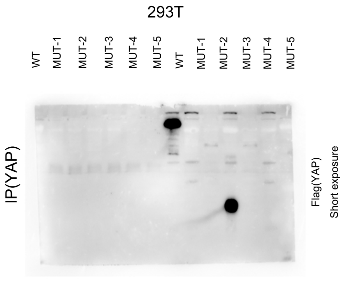

Supplement3 C

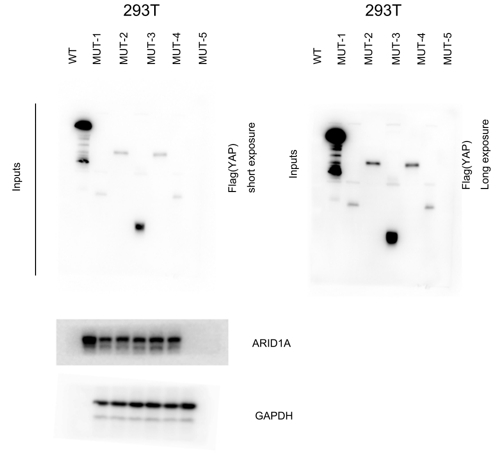

Supplement3 D

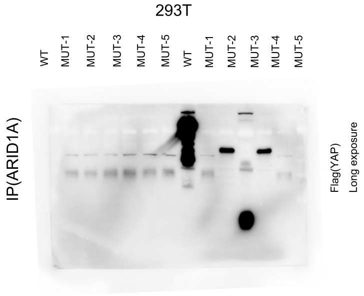

Supplement: Supplementary file 1 [file cancers-15-02447-s001.zip › cancers-2278291-File S1-original WB/S3.pdf]
