# Supplementary material for: Chromatin Remodelling Molecule ARID1A Determines Metastatic Heterogeneity in Triple-Negative Breast Cancer by Competitively Binding to YAP
Source: Cancers (Basel). 2023 Apr 25;15(9):2447. doi: 10.3390/cancers15092447 (PMC10177047; doi:10.3390/cancers15092447)
Supplement: Supplementary file 1 [file cancers-15-02447-s001.zip › cancers-2278291-supplementary figures.pdf]

Figure S1

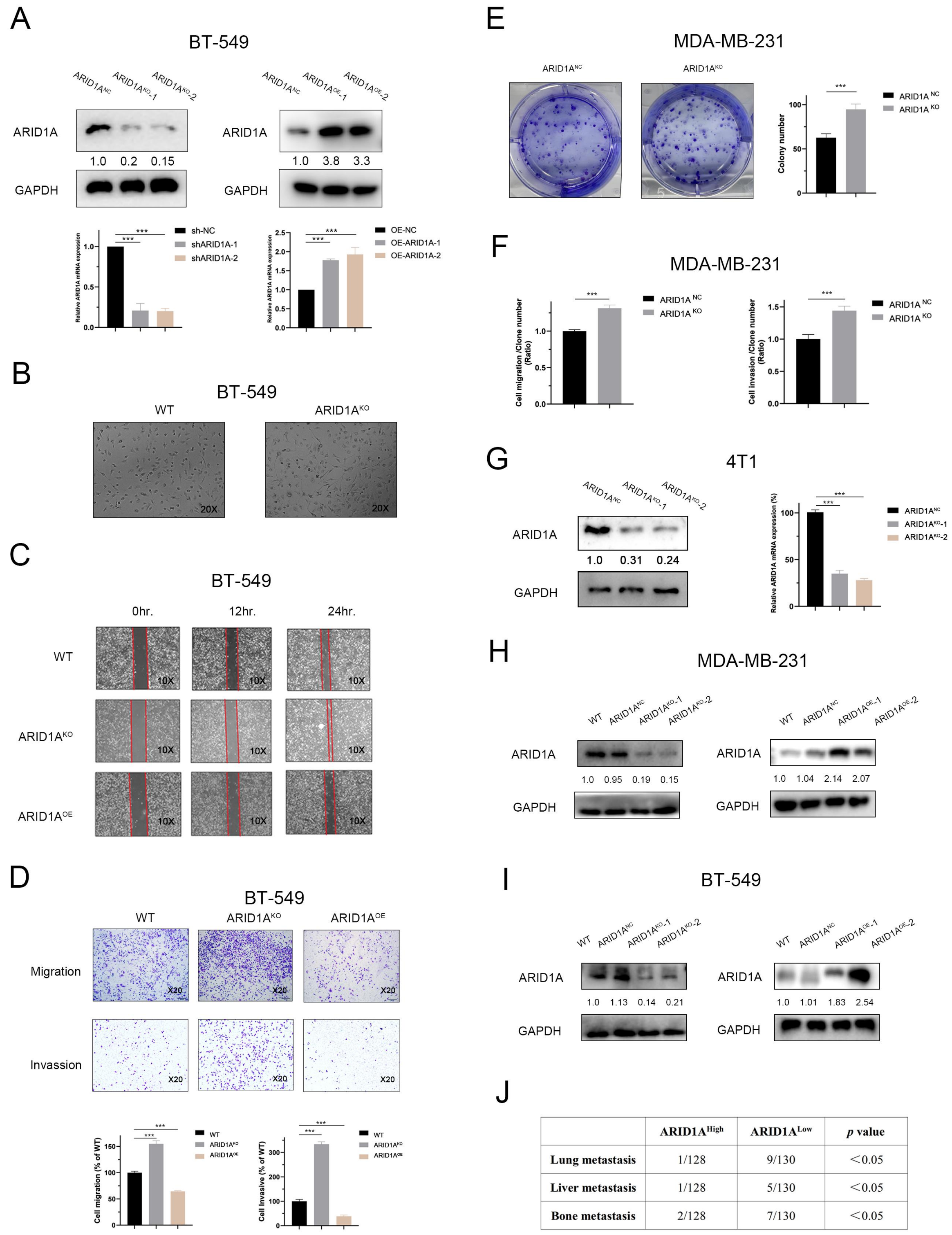

	ARID1A^{High}	ARID1A^{Low}	*p* value
**Lung metastasis**	1/128	9/130	<0.05
**Liver metastasis**	1/128	5/130	<0.05
**Bone metastasis**	2/128	7/130	<0.05

Figure S1: Inhibition of ARID1A promotes migration and invasion in TNBC cells. **(A)** BT-549 cells were silenced by CRISPR/Cas9-mediated knockdown and overexpressed by a plasmid. ARID1A expression levels were determined by Western blotting and qRT-PCR. **(B)** Cell morphology was observed under an optical microscope in BT-549 cells of each group. **(C-D)** Migration and invasion of BT-549 cells from each group were detected by wound healing assay **(C)** and transwell

migration and invasion assay **(D)**. **(E)** Cloning formation experiment in ARID1A^{NC} and ARID1A^{KO} cells. **(F)** Cell invasion and metastasis rate after standardised clonal growth. **(G)** 4T1 cells were silenced by CRISPR/Cas9-mediated knockdown. ARID1A expression levels were determined by Western blotting and qRT-PCR. **(H)** Western blot assay of ARID1A expression in MDA-MB-231 cells in the wild type, ARID1A^{NC}, ARID1A^{KO}, or ARID1A^{OE} groups. **(I)** Western blot assay of ARID1A expression in BT-549 cells in the wild type, ARID1A^{NC}, ARID1A^{KO}, or ARID1A^{OE} groups. **(J)** Statistics and analysis of lung, liver, and bone metastasis in 258 TNBC patients. Error bars represent the standard deviation among replicates. Data are presented as the mean ± SEM (n = 3), and statistical significance was determined by *** $p < 0.05$.

Figure S2

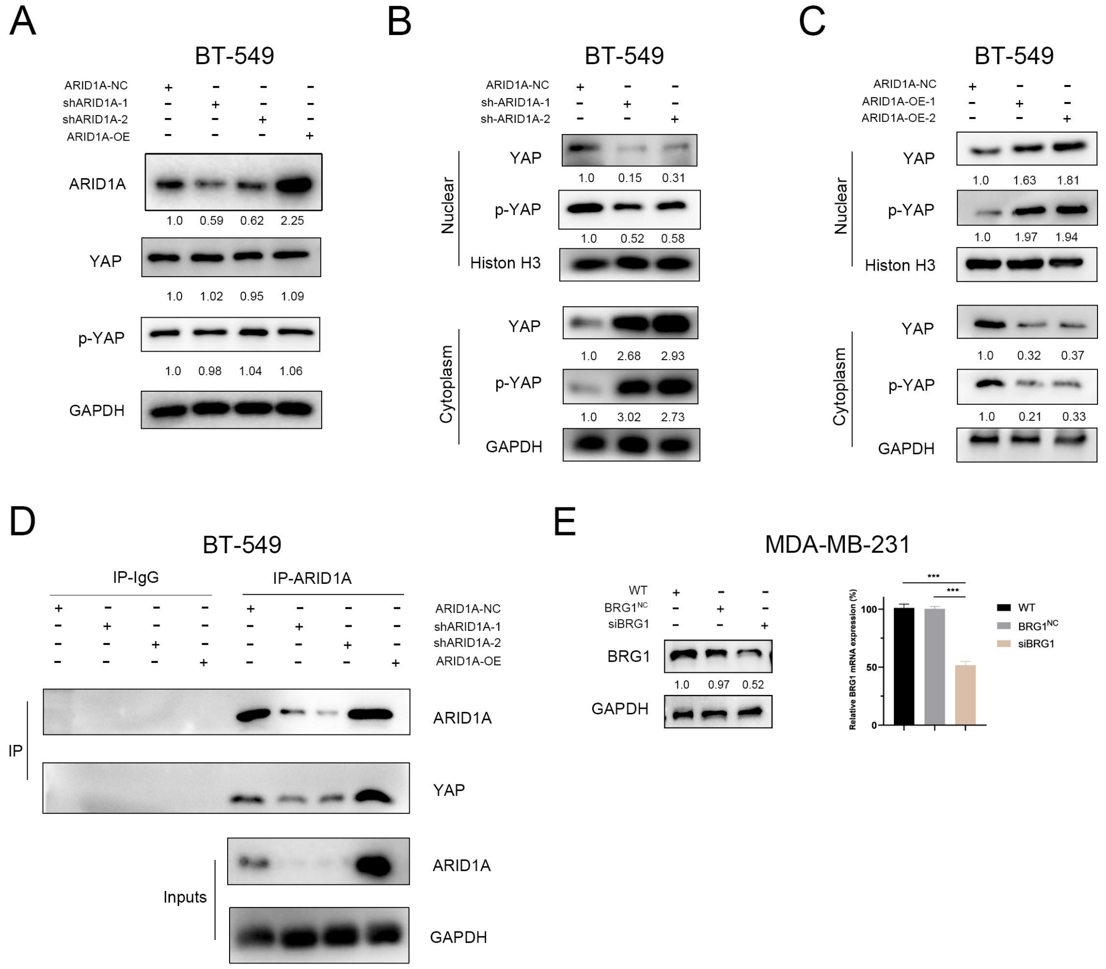

Figure S2: ARID1A competitively binds YAP to form an ARID1A/YAP complex. **(A)** Western blot assay of YAP expression in each group of BT-549 cells. **(B)** Western blot assay of cytoplasmic and nuclear YAP and p-YAP expression in MDA-MB-231 cells between ARID1A^{NC} and ARID1A^{KO} groups. **(C)** Western blot assay of cytoplasmic and nuclear YAP and p-YAP expression in MDA-MB-231 cells between ARID1A^{NC} and ARID1A^{OE} groups. Western blot assay of cytoplasmic and nuclear YAP expression in each group of BT-549 cells. **(D)** ARID1A and YAP interaction was detected in BT-549 cells using anti-ARID1A and anti-YAP antibodies for Co-IP and Western blot assays. **(E)** MDA-MB-231 cells were knocked down by siBRG1. BRG1 expression levels were determined by Western blotting and qRT-PCR. Error bars represent the standard deviation among replicates. Data are presented as the mean ± SEM (n = 3).

Figure S3

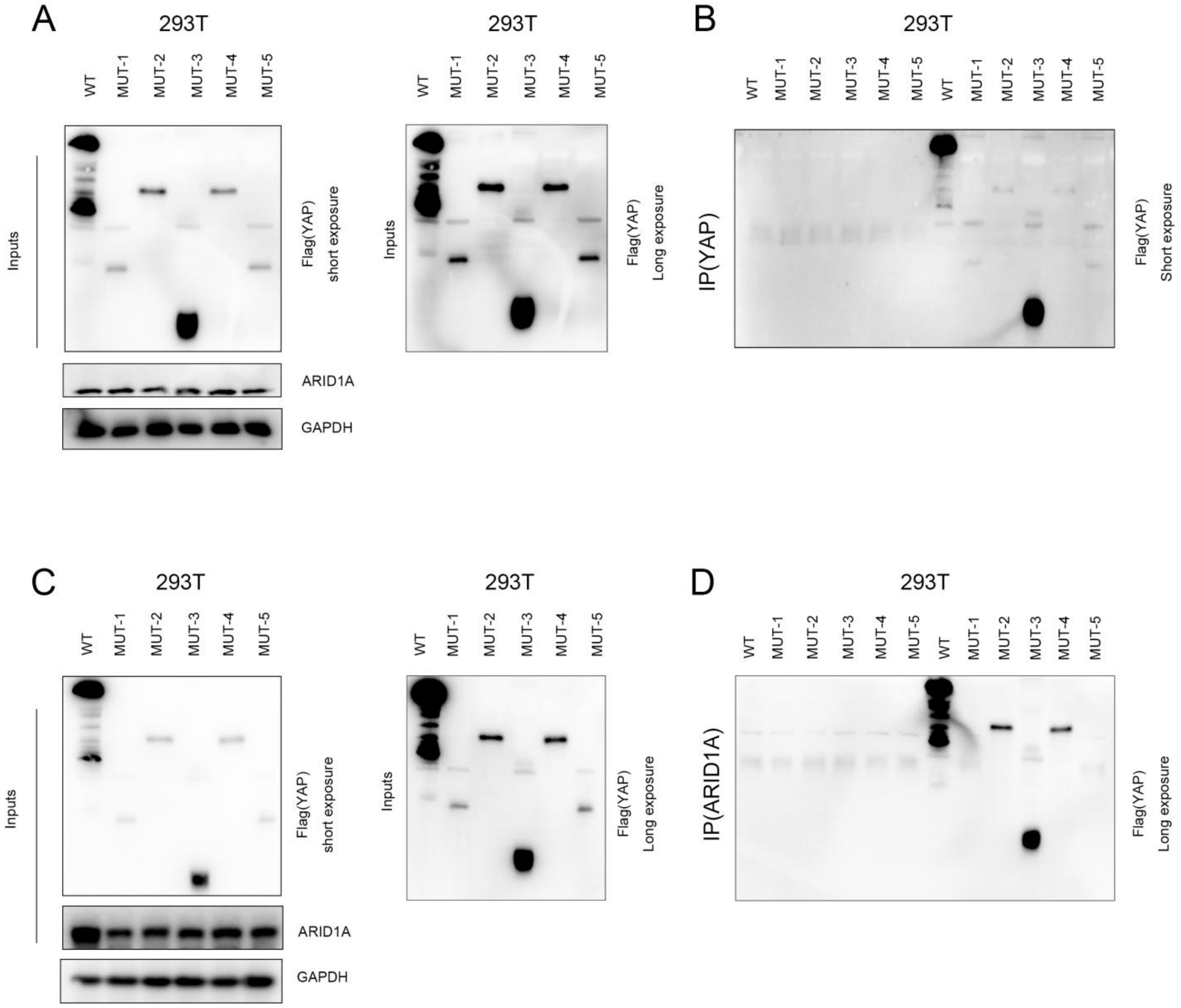

Figure S3: ARID1A binds with YAP via the WW domain. **(A-B**) HEK293T cells were transfected with the indicated plasmids. Western blot (Input) and Co-IP (IP-YAP) assays were performed to assess the interaction between ARID1A and truncated mutations of YAP. **(C-D**) HEK293T cells were transfected with the indicated plasmids. Western blot (Input) and Co-IP (IP-ARID1A) assays were performed to assess the interaction between ARID1A and truncated mutations of YAP. Error bars represent the standard deviation among replicates. Data are presented as the mean ± SEM (n = 3).
