# Supplementary material for: Chromatin Remodelling Molecule ARID1A Determines Metastatic Heterogeneity in Triple-Negative Breast Cancer by Competitively Binding to YAP
Source: Cancers (Basel). 2023 Apr 25;15(9):2447. doi: 10.3390/cancers15092447 (PMC10177047; doi:10.3390/cancers15092447)
Supplement: Supplementary file 1 [file cancers-15-02447-s001.zip › cancers-2278291-supplementary tables.pdf]

Table S1. Sequences of the primers and shRNA used for this study

The primers sequences were as follows:

Gene	Forward sequence 5’-3’	Reverse sequence 5’-3’
ARID1A	AAGCCACCAACTCCAGCATCCA	CGCTTCTGGAATGTGGAGTCAC
mARIDA	CTTCCCCAACCACCAGTACAA	CTGTGCGAAGGACGAAGAC
E-cadherin	GCCTCCTGAAAAGAGAGTGGAAG	TGGCAGTGTCTCTCCAAATCCG
N-cadherin	CCTCCAGAGTTTACTGCCATGAC	GTAGGATCTCCGCCACTGATTC
Snail	TGCCCTCAAGATGCACATCCGA	GGGACAGGAGAAGGGCTTCTC
GAPDH	GTCTCCTCTGACTTCAACAGCG	ACCACCCTGTTGCTGTAGCCAA
mGAPDH	AGGTCGGTGTGAACGGATTTG	GGGGTCGTTGATGGCAACA

The shRNA sequences were as follows:

Species	Gene	Sequence
mouse	ARID1A^{KO}-1	5′-GGTCCCTGTTGTTGCGAGTA-3′
mouse	ARID1A^{KO}-2	5′-gACCCCATGACCATGCAGGGC-3′
mouse	YAP^{KD}-1	5′-AGGCCAGTACTGATGCAGGTA-3′
mouse	YAP^{KD}-2	5′-CAGGACCTCTTCCTGATGGAT-3′
human	ARID1A^{KO}-1	5′-CACCGATGGTCATCGGGTACCGCTG-3′
human	ARID1A^{KO}-2	5′-CACCGCCCCTCAATGACCTCCAGTA-3′
human	ARID1A^{OE}-1	5′-CACCGGGCGCTCTAGCCGCTCAGTC-3′
human	ARID1A^{OE}-2	5′-CACCGCTTGGGTCGAGGCTGCTGCG-3′
human	YAP^{KD}-1	5′-CCCAGTTAAATGTTCACCAAT-3′
human	YAP^{KD}-2	5′-CACCAAGCTAGATAAAGAA-3′

Table S2: Characteristics of 258 patients in FUSCC TNBC cohort

Number	ARID1A rank	OS/m	RFS/m	0=survival，1=die
1	hi	56.1	56.1	0
2	low	21.0	21.0	0
3	low	48.7	48.7	0
4	hi	54.2	30.8	0
5	low	55.0	55.0	0
6	hi	60.5	30.5	0
7	hi	48.0	47.9	0
8	hi	58.3	30.7	0
9	low	16.6	8.8	0
10	low	53.7	53.7	0
11	low	62.9	48.7	0
12	hi	57.8	38.7	0
13	low	54.4	54.4	0
14	low	45.3	23.2	0
15	low	37.5	20.7	0
16	hi	37.7	37.7	0
17	hi	58.3	58.3	0
18	low	55.7	55.7	0
19	low	37.3	37.3	0
20	hi	55.9	55.9	0
21	low	45.6	45.6	0
22	hi	60.3	30.5	0
23	low	30.5	30.5	0
24	low	44.2	44.2	0
25	hi	55.2	55.2	0
26	hi	57.5	57.5	0
27	hi	56.8	56.8	0
28	low	57.0	57.0	0
29	hi	56.1	56.1	0
30	hi	48.3	30.5	0
31	low	37.3	33.4	0
32	hi	56.3	56.3	0
33	hi	60.9	30.8	0
34	hi	56.9	30.5	0
35	hi	53.7	53.7	0
36	hi	58.5	57.8	0
37	low	49.9	49.9	0
38	low	59.5	19.9	1

39	low	20.7	12.9	1
40	low	59.0	26.1	0
41	low	55.3	55.3	0
42	low	26.0	23.5	0
43	hi	42.2	20.7	1
44	low	56.4	56.4	0
45	low	43.8	27.7	0
46	low	49.2	30.4	0
47	low	52.9	30.5	0
48	hi	58.6	31.1	0
49	hi	58.1	58.1	0
50	low	60.2	30.4	0
51	low	60.4	38.3	0
52	low	55.4	55.4	0
53	low	47.0	47.0	0
54	hi	57.1	57.1	0
55	hi	57.9	57.9	0
56	low	58.2	30.3	0
57	low	32.9	24.5	1
58	hi	54.6	54.6	0
59	low	56.5	56.5	0
60	hi	61.6	30.1	0
61	hi	50.3	50.3	0
62	hi	54.6	54.6	0
63	hi	57.1	53.2	0
64	hi	58.1	58.1	0
65	hi	62.0	30.2	0
66	hi	57.9	57.9	0
67	hi	55.9	55.9	0
68	low	26.9	26.3	1
69	hi	47.5	47.5	0
70	hi	53.5	28.0	0
71	hi	56.5	56.5	0
72	hi	61.5	29.9	0
73	hi	57.1	57.1	0
74	hi	55.8	55.8	0
75	hi	57.4	57.4	0
76	hi	57.9	57.9	0
77	hi	43.4	30.2	0
78	hi	58.3	30.2	0
79	hi	56.4	56.4	0

80	low	58.0	30.2	0
81	low	57.4	30.4	0
82	low	54.3	54.3	0
83	hi	38.3	38.3	0
84	hi	53.3	53.3	0
85	low	14.8	6.2	0
86	low	57.8	57.8	0
87	hi	57.0	57.0	0
88	hi	36.7	24.4	0
89	hi	53.8	53.8	0
90	low	48.0	30.0	0
91	hi	54.6	54.6	0
92	low	30.0	30.0	0
93	low	59.8	29.8	0
94	hi	48.8	48.8	0
95	hi	54.1	54.1	0
96	low	5.8	5.8	0
97	hi	55.0	55.0	0
98	hi	58.1	57.4	0
99	hi	54.7	54.7	0
100	low	53.3	0.8	0
101	hi	55.1	48.7	0
102	low	37.5	37.5	0
103	low	55.9	55.9	0
104	hi	57.6	57.6	0
105	low	56.8	17.9	0
106	low	0.5	0.5	0
107	hi	58.3	53.1	0
108	hi	43.2	27.3	0
109	low	53.1	1.4	0
110	low	57.1	29.5	0
111	low	36.4	8.7	1
112	low	57.7	29.5	0
113	low	56.8	56.8	0
114	low	55.8	55.8	0
115	hi	57.9	29.7	0
116	low	56.0	46.6	0
117	low	27.5	27.5	0
118	low	55.8	55.8	0
119	hi	57.1	57.1	0
120	low	55.0	53.2	0

121	low	48.5	48.5	0
122	low	31.3	31.3	0
123	low	29.6	29.6	0
124	hi	57.9	29.7	0
125	hi	57.4	13.0	0
126	low	18.7	18.7	0
127	low	15.8	15.8	0
128	hi	44.5	44.5	0
129	low	58.5	10.0	0
130	hi	57.4	21.6	0
131	hi	56.7	56.7	0
132	low	52.9	29.5	0
133	low	53.6	53.6	0
134	hi	56.9	56.9	0
135	low	42.2	27.2	0
136	low	57.2	57.2	0
137	hi	48.1	48.1	0
138	low	62.3	0.7	0
139	low	35.1	35.1	0
140	low	36.1	22.4	1
141	hi	56.6	52.5	0
142	low	39.4	24.7	0
143	low	29.5	29.5	0
144	low	57.5	29.3	0
145	hi	40.2	40.2	0
146	hi	38.7	38.7	0
147	hi	56.5	56.5	0
148	low	46.1	23.3	0
149	hi	52.1	52.1	0
150	low	55.5	55.5	0
151	hi	54.5	54.5	0
152	hi	56.7	56.7	0
153	low	42.7	26.5	0
154	hi	54.5	54.5	0
155	hi	49.4	49.4	0
156	low	55.1	55.1	0
157	low	26.7	26.7	0
158	low	45.8	29.0	1
159	hi	54.0	54.0	0
160	hi	58.9	45.6	0
161	low	69.1	0.0	1

162	low	42.9	25.0	0
163	low	32.6	26.8	0
164	low	23.4	23.4	0
165	low	43.6	25.0	0
166	hi	33.5	26.0	0
167	low	52.1	3.1	0
168	low	39.2	25.2	0
169	low	25.9	24.9	0
170	hi	25.3	25.3	0
171	low	56.9	26.5	0
172	low	49.9	25.6	0
173	hi	38.0	25.6	0
174	low	39.0	25.3	0
175	low	40.5	26.0	0
176	low	50.8	12.8	0
177	low	37.8	25.7	0
178	low	44.3	25.6	0
179	hi	38.1	26.4	0
180	low	35.7	25.1	0
181	low	42.0	25.7	0
182	low	39.9	24.2	1
183	low	30.6	23.1	0
184	low	29.3	24.8	0
185	low	40.3	25.8	0
186	low	36.4	23.9	0
187	hi	38.0	13.8	0
188	low	38.1	24.7	0
189	low	44.3	26.3	0
190	low	50.6	25.7	0
191	hi	50.6	25.1	0
192	low	43.8	26.4	0
193	hi	42.0	26.8	0
194	hi	8.3	7.9	1
195	hi	56.8	27.4	0
196	low	40.6	25.2	0
197	hi	28.0	28.0	0
198	low	39.7	25.9	0
199	hi	44.3	28.8	0
200	low	39.3	25.9	0
201	hi	54.8	26.7	0
202	low	39.9	29.2	0

203	low	50.9	28.2	0
204	hi	55.6	29.4	0
205	hi	55.0	27.0	0
206	hi	56.0	56.0	0
207	low	55.1	55.1	0
208	low	40.9	13.1	1
209	hi	59.3	59.3	0
210	low	44.8	26.1	0
211	hi	59.3	59.3	0
212	hi	45.7	31.5	0
213	low	61.7	59.7	0
214	hi	60.4	59.5	0
215	hi	60.0	60.0	0
216	hi	59.5	31.4	0
217	low	42.4	32.1	0
218	hi	57.1	57.1	0
219	hi	84.7	49.3	1
220	hi	55.3	55.3	0
221	low	29.4	27.0	1
222	hi	55.1	55.1	0
223	low	24.7	13.2	1
224	hi	35.7	29.7	0
225	low	57.3	29.7	0
226	hi	60.4	59.5	0
227	hi	61.2	58.1	0
228	hi	60.4	59.5	0
229	low	45.9	23.0	1
230	hi	40.7	33.0	0
231	hi	59.9	26.1	0
232	hi	57.1	55.5	0
233	low	31.8	31.8	0
234	low	3.2	3.2	0
235	hi	62.4	62.4	0
236	hi	43.2	22.5	0
237	hi	61.4	61.4	0
238	hi	61.2	61.2	0
239	hi	46.7	33.4	0
240	hi	32.6	32.6	1
241	low	60.9	60.9	0
242	low	45.4	13.9	0
243	hi	61.2	61.2	0

244	hi	60.2	60.2	0
245	low	38.1	25.5	0
246	hi	60.9	60.9	0
247	low	41.1	11.5	1
248	low	61.4	61.4	0
249	hi	61.8	61.8	0
250	hi	60.5	60.0	0
251	hi	65.1	61.4	0
252	low	60.5	59.4	0
253	hi	59.4	59.4	0
254	hi	62.8	33.5	0
255	hi	59.8	59.8	0
256	low	24.8	24.8	0
257	hi	61.4	61.4	0
258	low	45.6	19.4	0
