# Supplementary figures and images for: Chromatin Remodelling Molecule ARID1A Determines Metastatic Heterogeneity in Triple-Negative Breast Cancer by Competitively Binding to YAP
Source: Cancers (Basel). 2023 Apr 25;15(9):2447. doi: 10.3390/cancers15092447 (PMC10177047; doi:10.3390/cancers15092447)

Fig 1F

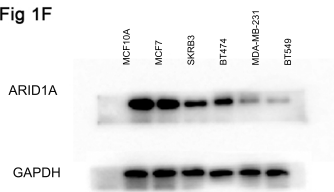

Fig 2A

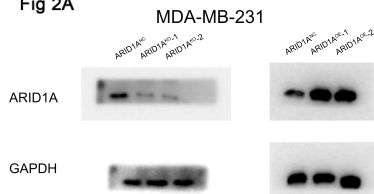

Fig 4A

MDA-MB-231

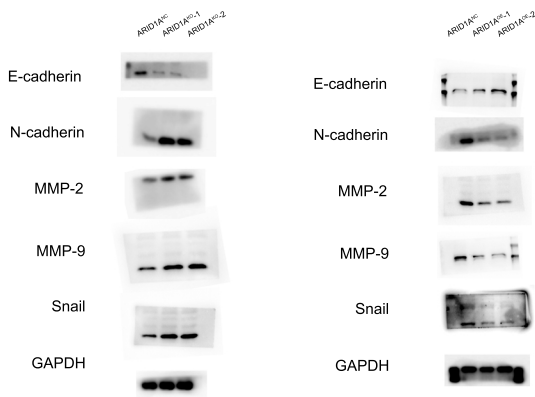

Fig 4B

BT-549

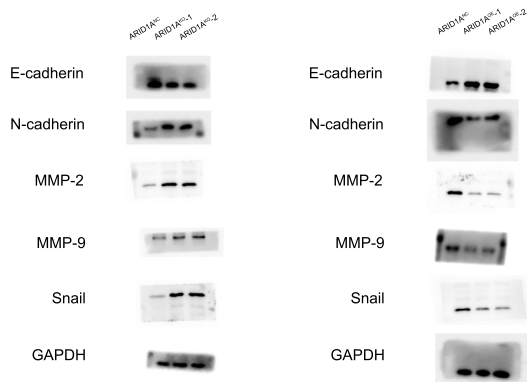

Supplement: Supplementary file 1 [file cancers-15-02447-s001.zip › cancers-2278291-File S1-original WB/1.pdf]

Fig 5D

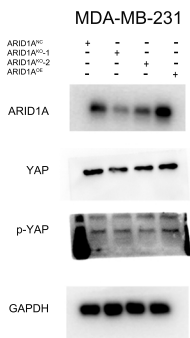

Fig 5E

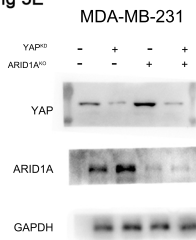

Fig 5H

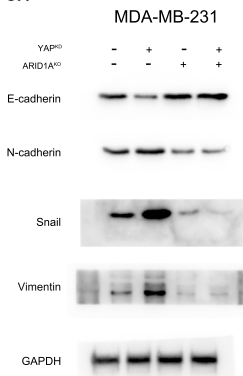

Supplement: Supplementary file 1 [file cancers-15-02447-s001.zip › cancers-2278291-File S1-original WB/2.pdf]

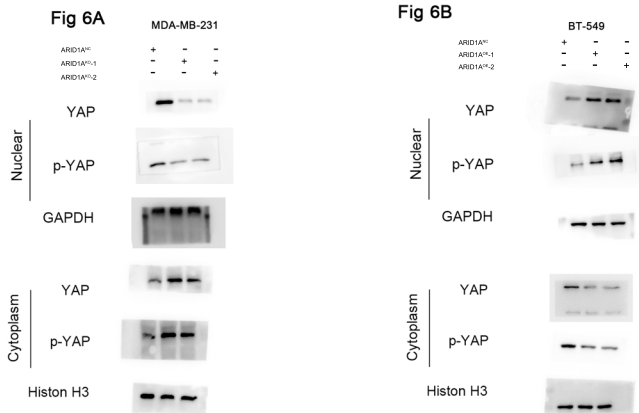

**Fig 6D**

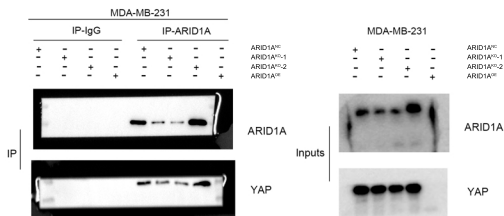

**Fig 6E**

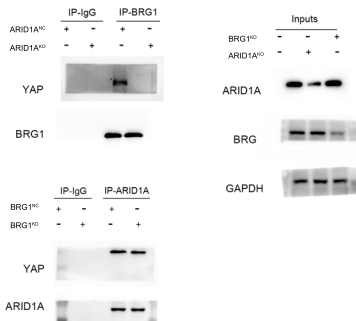

Supplement: Supplementary file 1 [file cancers-15-02447-s001.zip › cancers-2278291-File S1-original WB/3.pdf]

Fig 6F

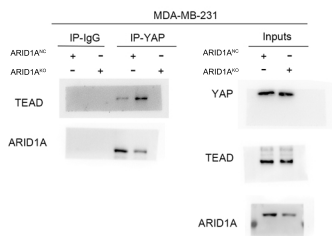

Fig7 B

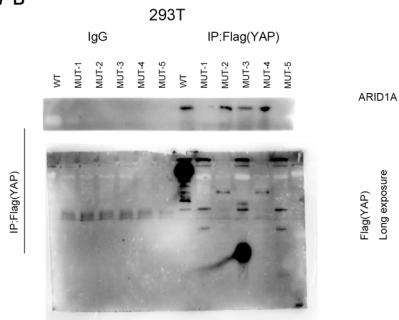

Fig7 C

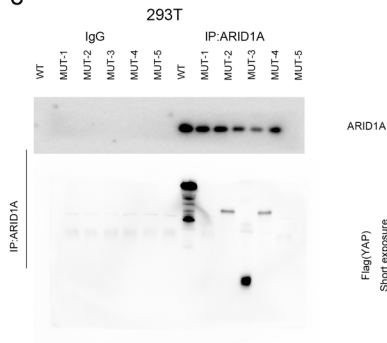

Fig F

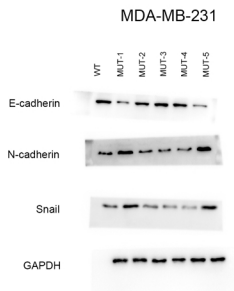

Supplement: Supplementary file 1 [file cancers-15-02447-s001.zip › cancers-2278291-File S1-original WB/4.pdf]

## Supplement1 A

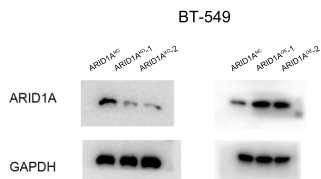

## Supplement1 G

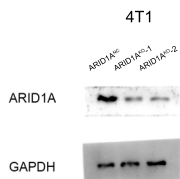

## Supplement1 H

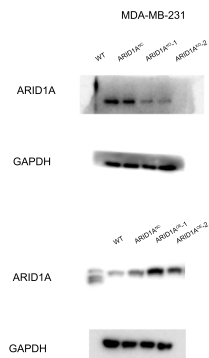

## Supplement1 I

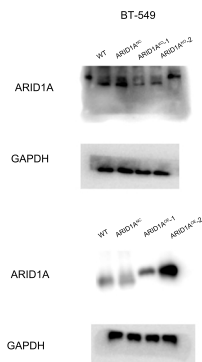

Supplement: Supplementary file 1 [file cancers-15-02447-s001.zip › cancers-2278291-File S1-original WB/S1.pdf]

Supplement2 A

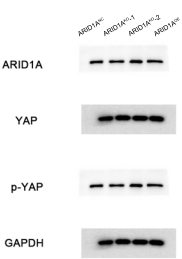

Supplement2 B

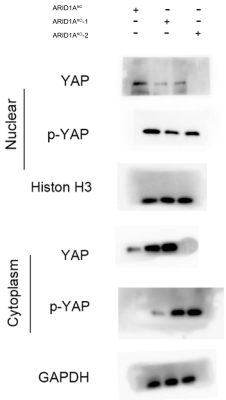

Supplement2 C

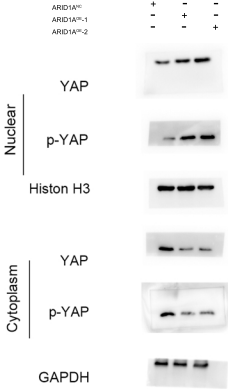

Supplement2 D

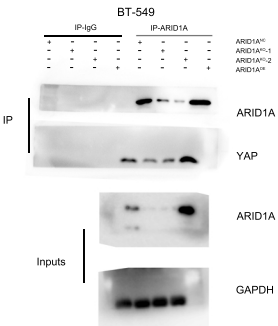

Supplement2 E

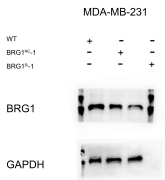

Supplement: Supplementary file 1 [file cancers-15-02447-s001.zip › cancers-2278291-File S1-original WB/S2.pdf]
